# MicroRNA-214 promotes hepatic stellate cell activation and liver fibrosis by suppressing Sufu expression

**DOI:** 10.1038/s41419-018-0752-1

**Published:** 2018-06-18

**Authors:** Liping Ma, Xiaoxue Yang, Rong Wei, Tinghong Ye, Jian-Kang Zhou, Maoyao Wen, Ruoting Men, Ping Li, Biao Dong, Lunxu Liu, Xianghui Fu, Heng Xu, Rami I. Aqeilan, Yu-Quan Wei, Li Yang, Yong Peng

**Affiliations:** 1 Department of Thoracic Surgery, State Key Laboratory of Biotherapy, West China Hospital, Sichuan University, and Collaborative Innovation Center for Biotherapy, 610041 Chengdu, China; 2 Department of Gastroenterology and Hepatology, West China Hospital, Sichuan University, 610041 Chengdu, China; 3 Department of Immunology and Cancer Research, Hebrew University-Hadassah Medical School, Jerusalem, Israel

## Abstract

MicroRNAs (miRNAs) have been demonstrated to modulate cellular processes in the liver. However, the role of miRNAs in liver fibrosis is poorly understood. Because the activation of hepatic stellate cells (HSCs) is a pivotal event in the initiation and progression of hepatic fibrosis, we investigate the differential expression of miRNAs in activated and quiescent rat HSCs by microarray analysis and find that miR-214 (miR-214-3p) is significantly upregulated during HSC activation. Moreover, the robust induction of miR-214 is correlated with liver fibrogenesis in carbon tetrachloride (CCl_4_)-treated rats and mice, high-fat diet-induced non-alcoholic steatohepatitis in mice, and cirrhosis in humans. We identify that miR-214 expression is driven by the helix–loop–helix transcription factor Twist1 via the E-box element. The increased miR-214 inhibits the expression of suppressor-of-fused homolog (Sufu), a negative regulator of the Hedgehog signaling pathway, thereby contributing to HSC activation to promote the accumulation of fibrous extracellular matrix and the expression of profibrotic genes in HSCs and LX2 cells. Furthermore, miR-214 expression is inversely correlated with the expression of Sufu in clinical cirrhosis samples. To explore the clinical potential of miR-214, we inject antagomiR-214 oligos into mice to induce hepatic fibrosis. The knockdown of miR-214 in vivo enhances Sufu expression and reduces fibrosis marker expression, which ameliorates liver fibrosis in mice. In conclusions, the Twist1-regulated miR-214 promotes the activation of HSC cells through targeting Sufu involved in the Hedgehog pathway and participates in the development of hepatic fibrosis. Hence, the knockdown of miR-214 expression may be a promising therapeutic strategy for liver fibrosis.

## Introduction

Many chronic liver diseases, such as hepatitis B or C infection, alcoholic liver disease, non-alcoholic steatohepatitis (NASH), and autoimmune hepatitis, can lead to hepatic fibrosis, which is characterized by the excessive accumulation of extracellular matrix (ECM) and disturbance of the lobular structure^1^. Hepatic stellate cells (HSCs) are the major producers of ECM, which play an important role in liver fibrogenesis. These cells are quiescent in healthy livers and are stimulated by profibrotic cytokines, including transforming growth factor-β (TGF-β), platelet-derived growth factor C (PDGF-C), and connective tissue growth factor^2, 3^. However, the mechanism underlying HSC activation is unclear. Understanding the mechanism of HSC activation is important for the development of effective anti-fibrotic therapies.

Multiple intracellular signaling pathways are involved in liver fibrogenesis. For example, TGF-β signaling pathway activates HSCs and promotes hepatic fibrosis through the phosphorylation of Smad family members^4, 5^. The PI3K-Akt signaling pathway is also critical for PDGF-induced HSC proliferation by modulating the expression of the type I collagen gene^6^. Recently, Hedgehog signaling pathway was found to activate HSCs for proliferation, indicating its important role in liver fibrosis^7, 8^. The Hedgehog pathway is activated when Hedgehog ligands bind to their receptor Patched (PTCH), leading to relief of Smoothened (SMO) from PTCH. Then, SMO translocates Gli family members (Gli1, Gli2, and Gli3) from the cytoplasm to the nucleus to regulate the expression of Hedgehog target genes^9^. Suppressor-of-fused homolog (Sufu) has been shown to interact with Gli transcription factors to inhibit their transcriptional activities, thus acting as a negative regulator of the Hedgehog pathway^10, 11^. Therefore, the downregulation of Sufu expression caused by different mechanisms probably participated in HSC activation and liver fibrosis.

MicroRNAs (miRNAs) are a class of small, noncoding RNAs with 21–23 nt in length, whose seed sequences are partially or completely complementary to target sequences within the 3′-untranslated region (UTR) of mRNAs, resulting in protein translation repression or target mRNA destabilization^12^. Increasing evidence demonstrates that miRNAs play a pivotal role in various physiological and pathological processes, including fibrosis and carcinogenesis^13–15^. For example, miR-29 expression is downregulated in human and murine liver fibrosis, which is mediated by TGF-β and other inflammatory signals^16^. miR-19b was found to inhibit TGF-β signaling, and its expression decreased in patients with advanced fibrosis, suggesting the potential of miR-19b as a therapeutic target for hepatic fibrosis^17^. Therefore, investigating how miRNAs are involved in HSC activation may expedite the discovery of new therapeutic targets and efficacious treatment strategies for hepatic fibrosis.

In this study, we compared miRNA expression profiles between activated and quiescent HSCs (qHSCs) using microarray analysis and identified miR-214 as a significantly upregulated miRNA during HSC activation. These findings were subsequently verified in animal models of liver injury and in clinical patient samples. Moreover, miR-214 expression is controlled by the transcription factor Twist1, which binds to E-box elements within the miR-214 promoter. Mechanistically, miR-214 enhances HSC activation by suppressing Sufu expression and thus regulates the Hedgehog signaling pathway. Importantly, in vivo administration of antagomiR-214 relieves liver fibrosis in mice. Therefore, miR-214 plays a pivotal role in liver fibrosis by regulating Hedgehog signaling and may be used as a potential therapeutic target.

## Results

### miR-214 expression is increased during HSC activation

**Fig. 1:**
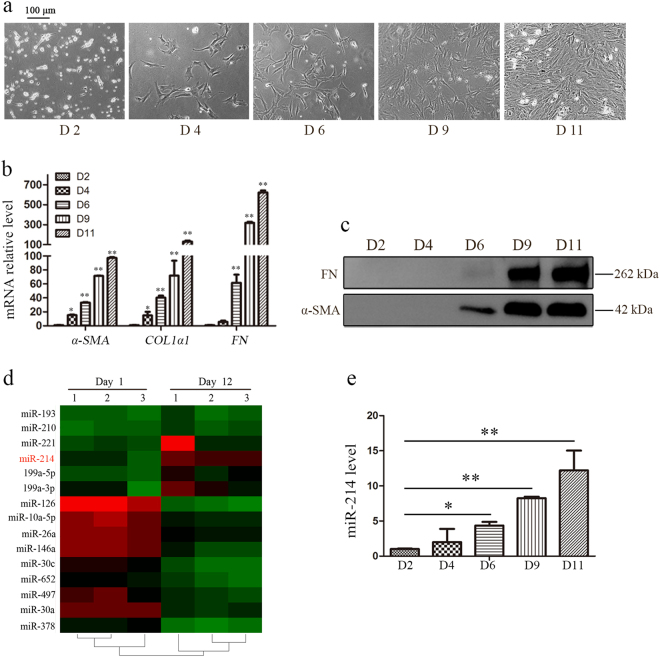
Increased expression of miR-214 in activated HSCs. **a** Morphology of primary rat HSCs at different time points in vitro culture at ×40 magnification. **b** Expression of profibrotic mRNAs including *α-SMA*, *COL1α1*, and *FN* and **c** protein level of α-SMA and FN was upregulated following HSC activation. **d** Microarray analysis for miRNAs expression was performed using total RNAs extracted from quiescent HSCs (D1) and activated HSCs (D12), *n* = 3 per group. Heat map representing two-way hierarchical clustering of differentially expressed miRNAs. **e** Differential expression of miR-214 in primary rat HSCs was validated using RT-qPCR. Error bars denote standard error of the mean. RT-qPCR was performed in duplicate and repeated three times. Values represent the means ± standard deviation (SD), *P < 0.05 and **P < 0.01

To identify the miRNAs involved in liver fibrosis, we isolated primary HSCs from normal rat livers and cultured them. The primary HSCs were activated during in vitro culture and were accompanied by distinct stellate morphological changes (Fig. 1a). The activation of HSCs was further confirmed by the increased mRNA abundance of HSC activation markers including α-smooth muscle actin (*α-SMA*), collagen type 1-α1 (*COL1α1*), and fibronectin (*FN*) (Fig. 1b) and upregulated protein levels of α-SMA and FN (Fig. 1c). To investigate the changes in miRNA expression profiles after HSC activation, miRNA microarray analysis was performed on total RNAs extracted from qHSCs and activated HSCs (aHSCs). A total of 15 miRNAs that were significantly differentially expressed after HSC activation are shown in Fig. 1d. Among these dysregulated miRNAs, miR-378 was downregulated while miR-221 expression increased in aHSCs compared to qHSCs, consistent with previous reports^18, 19^ and demonstrating the reliability of our microarray data. Intriguingly, miR-214 was one of the most significantly upregulated miRNA in aHSCs and its upregulation was further validated by real-time quantitative PCR (RT-qPCR) analysis (Fig. 1e). These results indicated an increasing expression of miR-214 in aHSCs and suggested a key role of miR-214 in HSC activation.

### miR-214 expression is upregulated in different liver injury models

**Fig. 2:**
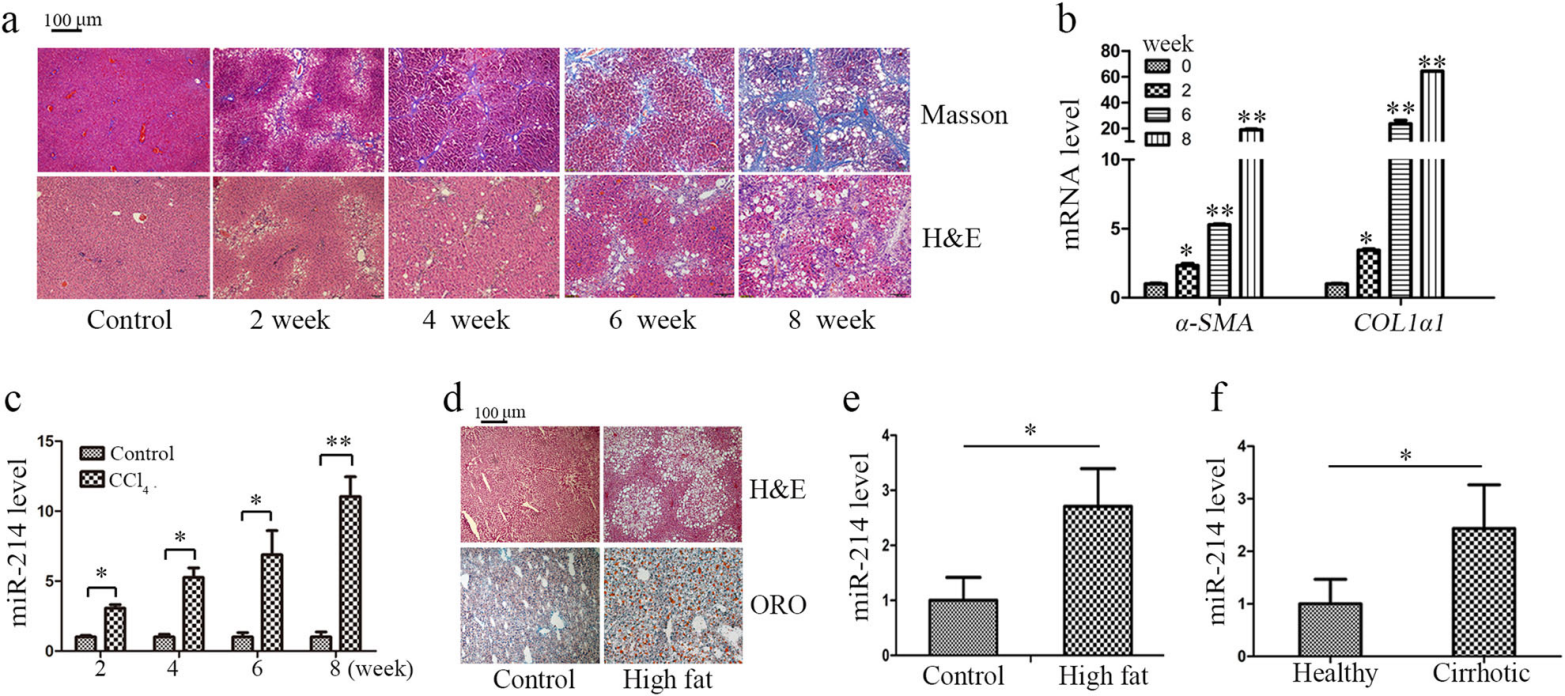
Upregulation of miR-214 in different liver injury models. **a** Liver fibrosis was induced by CCl_4_ injection for 2, 4, 6, or 8 weeks in rats (*n* = 6 per group). Representative images of H&E-stained and Masson’s trichrome-stained of rat liver sections at ×100 magnification. **b** The mRNA level of *α-SMA* and *COL1α1* and **c** miR-214 level was detected in CCl_4_-treated rat liver sections at different time points. **d** ORO-stained and H&E-stained images of HFD-treated mouse and control mouse liver at ×100 magnification (*n* = 3 per group). **e** miR-214 level was evaluated in liver samples from HFD-fed mice compared to control mice. **f** miR-214 expression in the liver of cirrhosis or healthy controls was measured by RT-qPCR. Relative expression levels are shown as the means ± standard deviation obtained from triplicate experiments (unpaired two-sample Student’s *t* test, *P < 0.05 and **P < 0.01)

In a systematic approach to identify the involvement of miR-214 in liver fibrosis, we employed a well-established rodent model of carbon tetrachloride (CCl_4_)-induced liver fibrosis^20^. To this end, we subcutaneously injected CCl_4_ into rats twice a week for 8 weeks. As control, the rats were injected with olive oil. Hematoxylin and eosin (H&E) staining of liver tissues showed that the number of apoptotic hepatocytes was increased accompanied by centrilobular necrosis in the livers of CCl_4_-treated rats, and Masson’s trichrome staining indicated that significant bridging fibrosis and fibrous septa were also observed in the liver sections from CCl_4_-treated rats (Fig. 2a). The severity of liver fibrosis depends on the length of CCl_4_ treatment time (Fig. 2a). With the prolongation of CCl_4_ treatment, the body weight of the rats were decreased, while the liver weight were increased (Supplementary Fig. 1a and 1b). Moreover, the mRNA level of *α-SMA* and *COL1α1* (Fig. 2b) and the protein level of FN were higher in the livers of CCl_4_-treated rats than those of control rats (Supplementary Fig. 1c and 1d). These results confirmed the successful induction of hepatic fibrosis in rats by CCl_4_ treatment. In this rat model of liver fibrosis, miR-214 expression was found to be upregulated in a manner dependent on the severity of liver fibrosis (Fig. 2c). Since NASH has been recognized as a major cause of liver fibrosis^21^, we examined miR-214 expression in the early stages of liver fibrosis. To this end, we induced NASH in mice by feeding them high-fat diet (HFD). The establishment of NASH was validated by H&E and Oil Red O (ORO) staining of liver sections, which revealed notable inflammation and lipid deposition (Fig. 2d). Compared with that in the control mice, miR-214 expression was significantly increased in the mice with HFD-induced liver samples (Fig. 2e), similar to what was observed in the rat model of CCl_4_-induced liver fibrosis. Advanced liver fibrosis causes cirrhosis and liver failure^22^. miR-214 expression was found to be significantly higher in human cirrhotic liver samples (Fig. 2f), and this was accompanied by high levels of FN protein (Supplementary Fig. 1e and 1f). Therefore, miR-214 likely plays a crucial role in the initiation or/and progression of liver fibrosis. However, further studies are needed to elucidate the overall function of miR-214 and the associated mechanisms in liver fibrosis.

### miR-214 promotes expression of profibrotic markers and cell proliferation in HSCs and LX2 cells

To study the functional relevance of miR-214 in fibrogenesis, we knocked down the expression of miR-214 using antagomiRs or overexpressed miR-214 using mimics in activated rat primary HSCs and human HSC cell line (LX2).

**Fig. 3:**
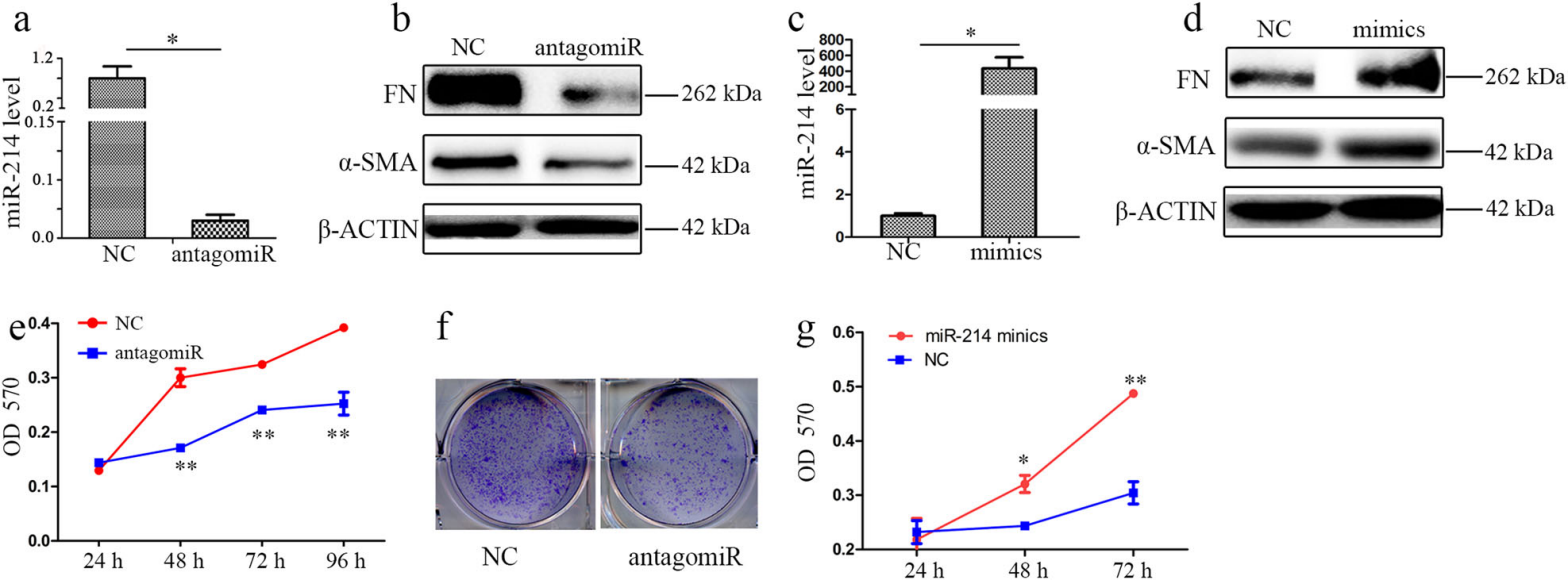
miR-214 regulates fibrotic gene expression and promotes HSC cell proliferation. **a** HSCs were transfected with either antagomiR-214 or NC-miR for 48 h, and the expression of miR-214 was detected by RT-qPCR and **b** protein levels of FN and α-SMA were examined using Western blotting. **c** HSCs were transfected with miR-214 mimics or NC-miR for 48 h, and the expression of miR-214 was detected by RT-qPCR and **d** the protein levels of FN and α-SMA were detected by Western blotting. **e** Proliferation of HSCs after transfection with antagomiR-214 or NC-miR was assessed using the MTT and **f** colony formation assays. **g** Proliferation of HSCs after transfection with miR-214 mimics or NC-miR was assessed using the MTT assay. Relative expression levels are shown as the means ± s.e.m obtained from triplicate experiments (unpaired two-sample Student’s *t* test, *P < 0.05 and **P < 0.01)

For the miR-214 knockdown study, HSCs and LX2 cells were transfected with antagomiR-214 or negative control (NC). As expected, miR-214 expression was successfully reduced by antagomiR-214 in HSCs (Fig. 3a). The protein level of FN and α-SMA were significantly repressed following the inhibition of miR-214 in HSCs (Fig. 3b). In contrast, the overexpression of miR-214 via mimics increased the protein level of FN and α-SMA (Fig. 3c, d). Similar results were obtained in human LX2 cells by the knockdown (Supplementary Fig. 2a and 2b) or overexpression of miR-214 (Supplementary Fig. 2d and 2e). These results demonstrated the functional relevance of miR-214 in fibrogenesis in vitro. Furthermore, cell growth was suppressed after the repression of miR-214 by antagomiR-214 in rat primary HSCs (Fig. 3e, f) and human LX2 (Supplementary Fig. 2c), which was accompanied by the decrease of cell numbers and the change of cell morphology (Supplementary Fig. 2g and 2h) in HSCs and LX2, respectively. In contrast, overexpression of miR-214 could significantly promote cell growth in HSCs (Fig. 3g) and LX2 cells (Supplementary Fig. 2f). Following the observation of miR-214-mediated growth promotion, we investigated whether miR-214 affects the cell cycle. Indeed, knockdown of miR-214 significantly increased the percentage of cells in the G1 phase and reduced the percentage of cells in S and G2 phases, indicating that miR-214 can influence cell cycle distribution in HSCs (Supplementary Fig. 2i–k).

### miR-214 regulates Sufu expression by direct binding to the 3′-UTR of its mRNA

**Fig. 4:**
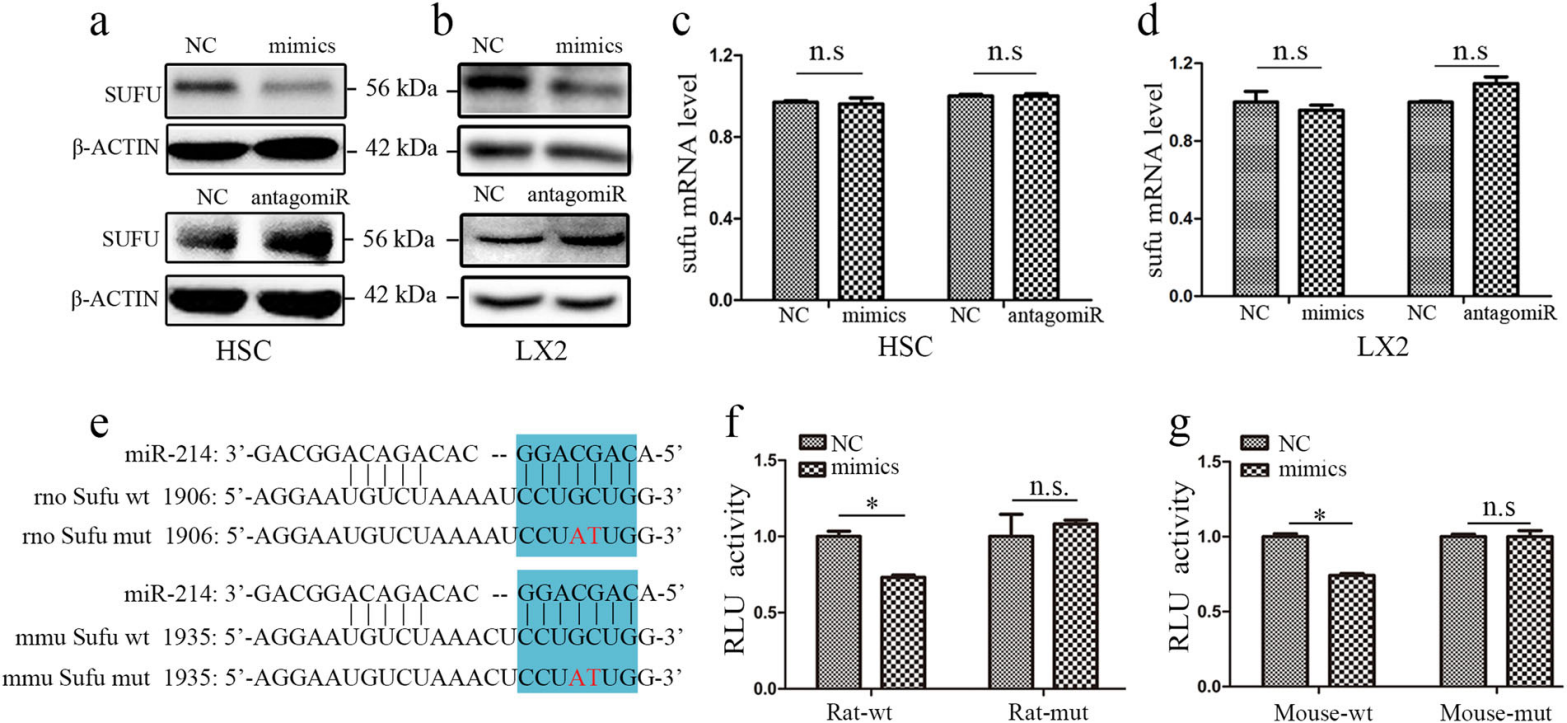
Sufu is a direct target of miR-214. **a**, **b** Inverse correlation between miR-214 and Sufu expression in (**a**) rat primary HSCs and **b** human LX2 cells transfected with miR-214 mimics or antagomiR-214. **c**, **d** RT-qPCR analyses of Sufu mRNA levels after transfection of miR-214 mimics or antagomiR in HSCs (**c**) and in LX2 cells (**d**). **e** Potential miR-214 binding sites were predicted in the 3′-UTR of rat or mouse Sufu mRNAs. Blue shading indicates the seed sequence of miR-214. **f**, **g** Dual-luciferase reporter assay was performed to verify the binding between (**f**) miR-214 and rat Sufu mRNA or (**g**) miR-214 and mouse Sufu mRNA. HEK 293T cells were co-transfected with vectors containing either the wild-type (wt) or mutant (mut) target sites of Sufu and either miR-214 mimics or negative control (NC). Relative luciferase activities (RLUs) were shown as the means ± S.E.M. obtained from triplicate experiments (two-sample Student’s *t* test, *P < 0.05 and **P < 0.01). n.s. nonsignificant

To explore the mechanism by which miR-214 regulates HSC activation and to identify the relevant target genes of miR-214, we conducted bioinformatics analyses using the commonly used software including TargetScan, miRBase, and miRanda, and found that Sufu, a downstream factor of Hedgehog signaling, could be a potential target of miR-214. Moreover, miR-214 has been reported to target the 3′-UTR of Sufu mRNA in human lung adenocarcinoma cells by luciferase assay^23^. However, whether miR-214 regulates Sufu expression in HSCs and in liver fibrosis models has not yet been examined. To this end, we transfected miR-214 mimics or antagomiR-214 into rat primary HSCs and human LX2 cells. As shown in Fig. 4a, b, Sufu expression clearly decreased in the cells transfected with miR-214 mimics compared with that in the control cells, while transfection with antagomiR-214 increased Sufu protein expression. However, qRT-PCR analysis demonstrated negligible effects of miR-214 on Sufu mRNA level (Fig. 4c, d), indicating that miR-214 may repress Sufu expression at the translational level. The sequence of mature miR-214 is identical in human, rat, and mouse, and bioinformatics analysis revealed that Sufu mRNAs have the potential miR-214 binding sites (Fig. 4e). To investigate whether miR-214 directly target Sufu expression, we cloned the 3′-UTR sequences of rat or mouse Sufu mRNA into the luciferase reporter vector. Moreover, we conducted sited-directed mutagenesis to destroy the potential miR-214 binding site to further validate target specificity (Fig. 4e). Luciferase activity was significantly repressed by the constructs harboring the wild-type rat or mouse miR-214 target sequence. Conversely, the constructs with the mutated forms of 3′-UTR resulted in no significant change in luciferase activity in HEK 293T cells (Fig. 4f, g) or HSCs (Supplementary Fig. 3a and 3b), indicating that miR-214 influences Sufu expression by directly targeting the 3′-UTR of Sufu mRNA.

### miR-214-mediated fibrogenesis depends on Sufu expression

**Fig. 5:**
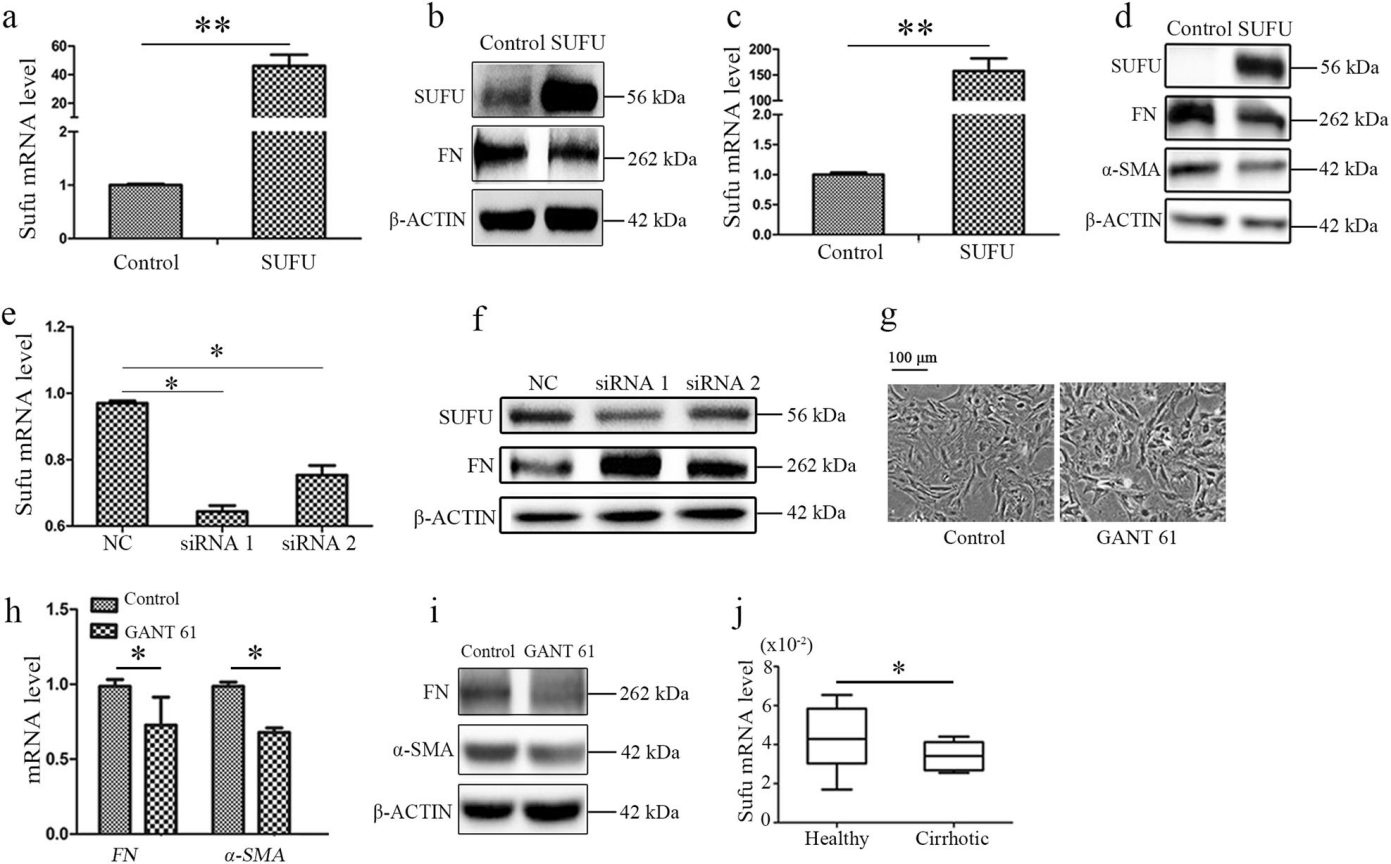
miR-214-mediated fibrogenesis depends on Sufu expression. **a** Overexpression of Sufu by lentivirus vector in HSCs and *SUFU* mRNA expression was measured by RT-qPCR and **b** the protein level of SUFU and FN was measured by Western blotting. **c** Overexpression of Sufu by lentivirus vector in LX2 and *SUFU* mRNA expression was measured by RT-qPCR and **d** the protein level of SUFU, FN, and α-SMA were measured by western blotting. **e** Knockdown of Sufu expression by siRNAs in HSCs and **f** the protein expression of SUFU and FN was measured by Western blotting. **g** Rat primary HSCs were treated with GANT-61 for 24 h and the fold changes in the profibrotic markers FN and α-SMA was detected by (**h**) RT-qPCR or (**i**) Western blotting. **j** The mRNA expression of *SUFU* in patients with cirrhosis or healthy control was measured by RT-qPCR. All results of relative expression values are shown as mean ± s.e.m. obtained from triplicate experiments (unpaired two-sample Student’s *t* test, *P < 0.05 and **P < 0.01)

Based on the above data, we hypothesized that miR-214 affects fibrogenesis by regulating Sufu expression. To verify our hypothesis, we used lentivirus to overexpress Sufu which resulted in downregulation of profibrotic markers expression in HSCs (Fig. 5a, b) and LX2 (Fig. 5c, d). Moreover, when Sufu expression was knocked down by small interfering RNA (siRNAs) in HSCs, the expression of FN protein was significantly upregulated (Fig. 5e, f). Sufu is known to physically interact with Gli transcription factors, which are critical regulators of the Hedgehog signaling pathway. When Sufu is overexpressed in the cells, it negatively affects the expression of Hedgehog target genes by inhibiting the transcriptional activities of Gli family members^10, 11^. To determine whether the Sufu downstream effector Gli was involved in fibrogenesis, we treated HSCs with GANT-61, an inhibitor of Gli transcriptional activity in the nucleus. The morphology of HSCs in the GANT-61-treated group was altered compared with that in the control group (Fig. 5g). Moreover, both mRNA and protein levels of FN and α-SMA were decreased in response to GANT-61 treatment (Fig. 5h, i), indicating that Gli transcription factors are involved in the regulation of fibrogenesis in vitro.

To further elucidate whether the effect of Sufu on hepatic fibrosis is clinically relevant, we examined the expression of Sufu in clinical cirrhosis samples. Indeed, Sufu expression was reduced in the clinical cirrhosis tissues (Fig. 5j) and was negatively correlated with miR-214 expression, indicating that miR-214 regulates fibrogenesis by targeting Sufu to modulate the Hedgehog signal pathway.

### Twist1 acts on an E-box elements to promote miR-214 expression in HSCs and LX2 cells

**Fig. 6:**
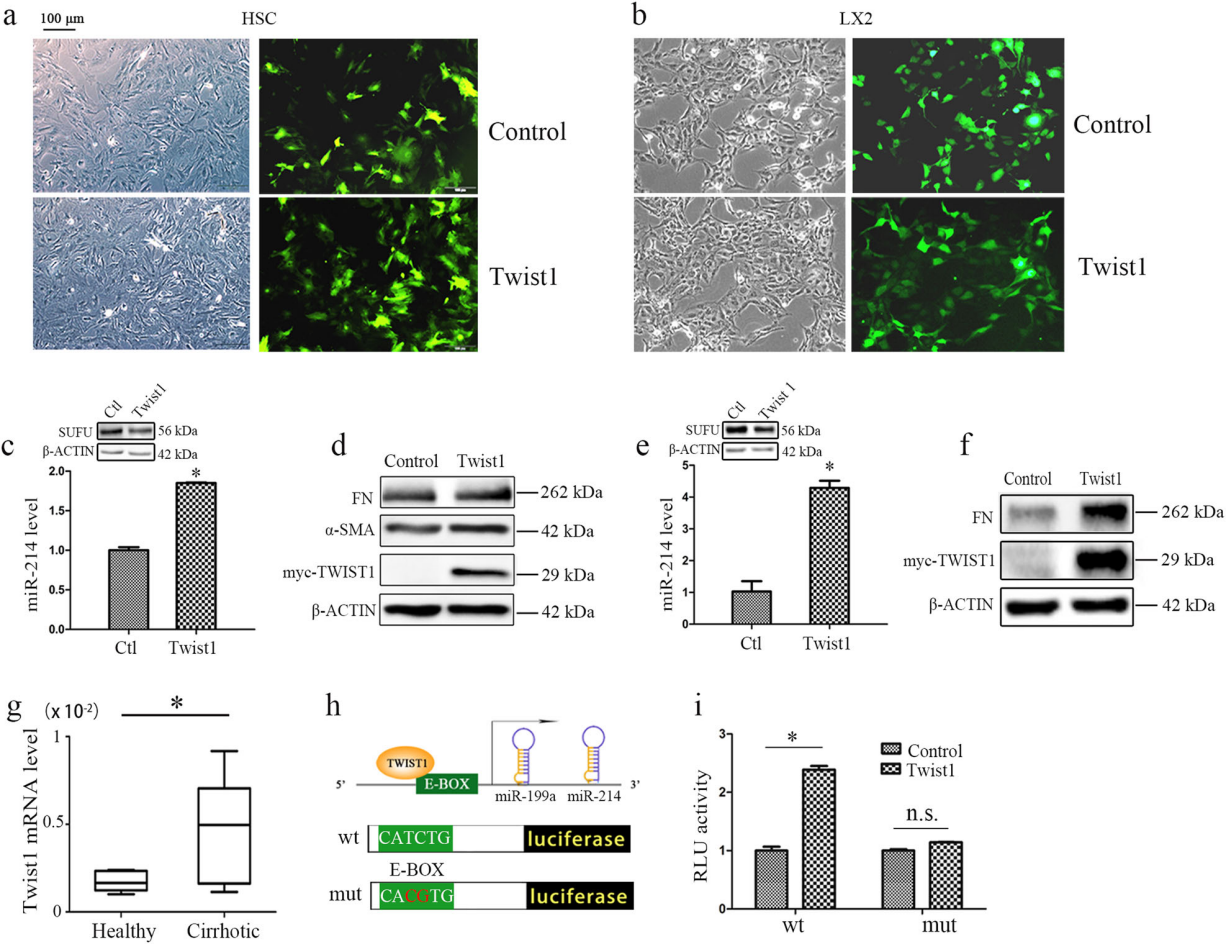
Twist1 affects liver fibrogenesis by regulating miR-214. **a**, **b** Twist1 overexpressing by lentiviral vectors containing GFP, fluorescence images for GFP expression (**a**) in HSCs, and (**b**) LX2 cells at ×100 magnification. **c** Twist1 overexpression upregulated miR-214 expression and downregulated SUFU expression, and (**d**) the protein levels of profibrotic markers FN and α-SMA was upregulated by Twist1 in HSCs. **e** Twist1 overexpression upregulated miR-214 expression and downregulated SUFU expression, and **f** the protein level of profibrotic markers FN was upregulated by Twist1 in LX2. **g** The expression of Twist1 in cirrhosis samples or healthy controls was measured by RT-qPCR. **h** Schematic representation of luciferase reporter constructs harboring wild-type or mutated proximal miR-214 promoter region and **i** relative luciferase activity (RLU) was measured. Relative expression levels are shown as the means ± s.e.m. obtained from triplicate experiments (unpaired two-sample Student’s *t* test, *P *<* 0.05). n.s. nonsignificant

The miR-199a/214 gene cluster is located in the Dynamin-3 (*DNM3*) gene as two clustered miRNAs approximately 6 kb apart. Twist1 has previously been reported to drive the expression of a single noncoding transcript from this gene locus^24^. Twist1 is a highly conserved transcription factor belonging to the family of basic helix–loop–helix proteins. To determine whether Twist1 regulates miR-214 expression in HSCs and in liver fibrosis models, we overexpressed Twist1 in rat HSCs (Fig. 6a) or LX2 (Fig. 6b) using lentiviral vectors containing GFP. As expected, Twist1 overexpression resulted in a significant increase in miR-214 expression accompanied by a reduction in Sufu expression in HSCs (Fig. 6c) or LX2 cells (Fig. 6e). Moreover, the expression of fibrosis markers FN and α-SMA was significantly increased in the Twist1-overexpressing group in HSCs (Fig. 6d) or LX2 cells (Fig. 6f). This indicated that Twist1 regulates the expression of miR-214, which indirectly affects Sufu levels and profibrotic markers expression in vitro. Furthermore, we observed high expression of Twist1 in clinical cirrhosis samples (Fig. 6g), which was positively correlated with miR-214 expression.

To further confirm that miR-214 expression is driven by Twist1, we analyzed miR-214 promoter sequences and noted that the promoter contains E-box elements, which are known to be bound by Twist1 homodimers^24^. Therefore, we amplified this region by PCR and cloned it next to the luciferase reporter gene. Additionally, we also constructed the mutation within the E-box element from CATCTG to CACGTG to destroy the function of E-box element as control (Fig. 6h). Indeed, luciferase activity was significantly increased in the cells transfected with the wild-type E-box construct. However, there was no change in the cells transfected with the mutant E-box construct (Fig. 6i). This confirmed the regulation of miR-214 expression by Twist1 via the E-box element.

### AntagomiR-214 ameliorates liver fibrosis induced by CCl_4_ in mice

**Fig. 7:**
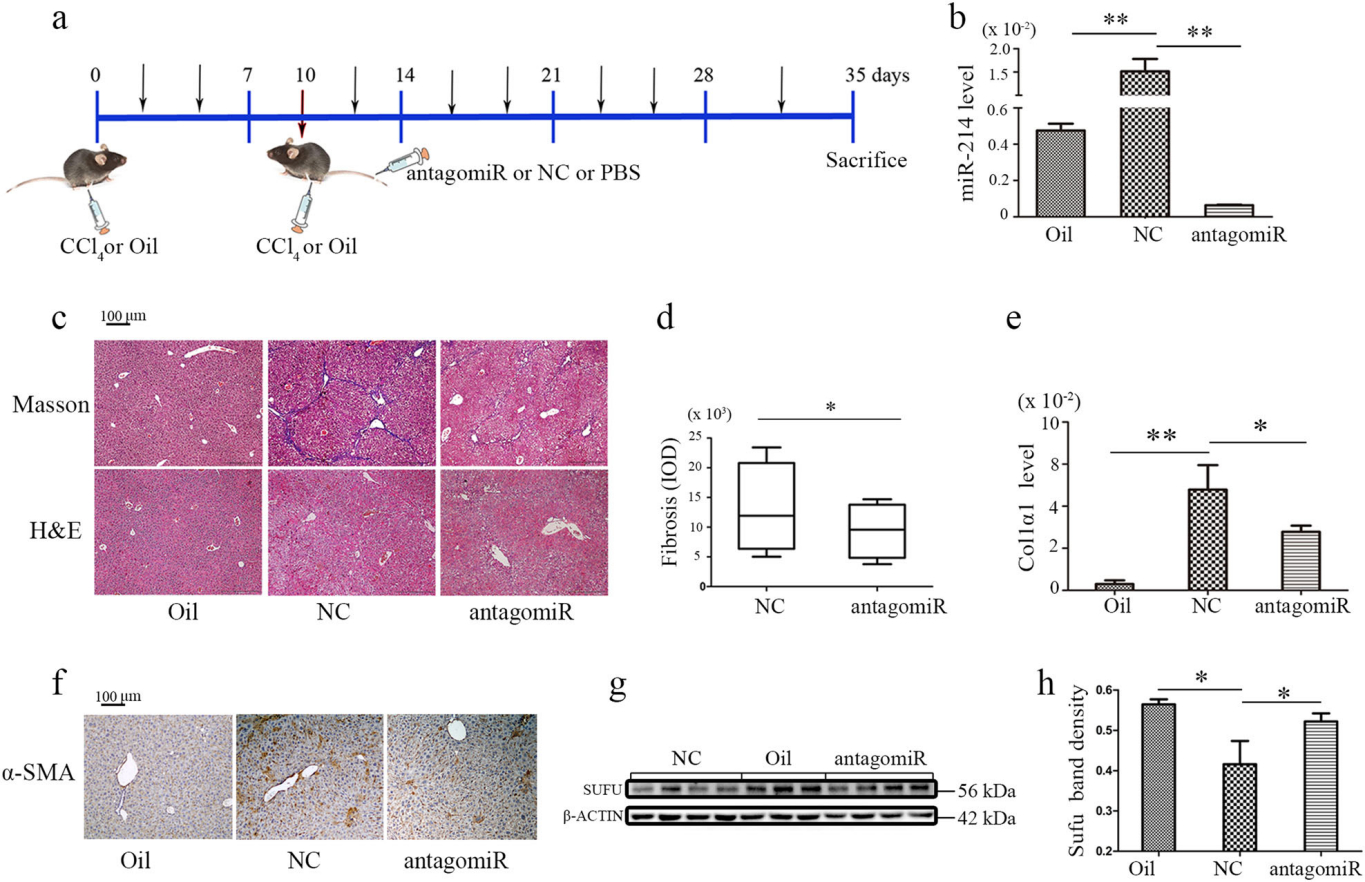
AntagomiR-214 ameliorates liver fibrosis in CCl_4_-treated mice by targeting Sufu. **a** Schema of the mice experimental model. **b** RT-qPCR data for miR-214 expression in livers from olive oil-treated, CCl_4_/NC-miR-treated, CCl_4_/antagomir-214-treated mice (*n* = 5 per group). **c** H&E and Masson’s trichrome staining in liver sections from representative mice from each group at ×100 magnification and **d** Masson-stained fibrosis areas were quantified using the ImagePro Plus software. **e** RT-qPCR analysis of *COL1α1* expression in the livers from mice of each group. **f** Representative immunohistochemistry images of α-SMA expression in the mouse livers from each group at ×100 magnification. **g** Western blot analysis for SUFU and internal control in the livers of representative mice from each group and **h** cumulative densitometric analyses of SUFU expression using Western blotting. Relative expression levels are shown as the means ± s.e.m. obtained from triplicate experiments (unpaired two-sample Student’s *t* test, *P *<* 0.05 and **P *<* 0.01)

According to the above data, miR-214 plays a vital role in fibrogenesis in vitro. Therefore, to confirm whether antagomiR-214 has a role in the prevention or treatment of hepatic fibrosis in vivo, we used mice that were intraperitoneally injected with CCl_4_ or olive oil twice weekly for 5 weeks to obtain mice with liver fibrosis or control mice, respectively. Treatments were initiated at day 10 after CCl_4_ injections. The animals received NC-miR or antagomiR-214 twice a week at a dose of 62.5 nmol via tail vein injection and mice were sacrificed after 5 weeks of treatment, and the livers were collected for further analysis (Fig. 7a). The level of miR-214 in liver tissues was examined via RT-qPCR. CCl_4_ treatment induced miR-214 overexpression in mouse livers, which was markedly suppressed by antagomiR-214 (Fig. 7b). Both Masson’s trichrome and H&E staining demonstrated the presence of hepatic injury and hepatic fibrosis in CCl_4_/NC group mice. However, in accordance with miR-214 reduction, liver damage was obviously ameliorated in the CCl_4_/antagomiR-214 group mice (Fig. 7c, d), which was accompanied by a reduction in *COL1α1* mRNA level (Fig. 7e) and decreased α-SMA levels, which as detected by immunohistochemistry (Fig. 7f). Importantly, Sufu expression was upregulated in response to antagomiR-214 treatment (Fig. 7g, h), suggesting that miR-214 suppression can modulate the expression of Sufu for the treatment of liver fibrosis and indicating miR-214 has a potential to be used as a therapeutic target in clinical studies for liver fibrosis.

## Discussion

Hepatic fibrosis results from chronic damage to the liver in accompany with the accumulation of ECM proteins, which is a progressive disease resulting in the development of cirrhosis and hepatocellular carcinoma^1^. Recent reports have shown that altered miRNA levels are also associated with the HSC activation and liver fibrosis^25^. However, the underlying mechanisms have not been clearly elucidated.

Whether miR-214 expression increases or decreases during HSC activation is controversial. Both Lakner et al.^17^ and Maubach et al.^26^ took advantage of microarray technology to identify that miR-214 is upregulated during HSC activation, whereas Chen et al.^27^ showed that miR-214 is downregulated. In this study, we found that miR-214 was the most significantly upregulated miRNA during HSC activation (Fig. 1). To further understand the differences, we found that Chen et al.^27^ normalized miR-214 expression to GAPDH mRNA while we normalized miR-214 levels to U6 RNA, a noncoding small nuclear RNA commonly used for miRNA measurement. Moreover, we also showed that the expression of miR-214 was upregulated with the progression of hepatic fibrosis in CCl_4_-treated rat or mouse model, an HFD-induced NASH mouse model and in patients with cirrhosis (Fig. 2). These results suggest that miR-214 may play a crucial role in the HSC activation and liver fibrosis. Through luciferase assay and RT-qPCR, we demonstrated that Twist1 bound to the E-box element within the promoter and induced miR-214 expression (Fig. 6). Twist1 was found to be upregulated during fibrosis^28, 29^, which further supports that miR-214 expression increased during liver fibrosis. The miR-199a/214 gene cluster is located in the 7.9-kb noncoding intron of the DNM3 gene. Twist1 has been reported to regulate the expression of the miR-199a/214 cluster during development^24^. Therefore, we overexpressed Twist1 to verify whether Twist1 also regulates miR-214 expression in HSCs. As expected, we found Twist1 induced the expression of miR-214 in HSCs and LX2 cells, respectively (Fig. 6c–f). Moreover, overexpression of Twist1 significantly increased the expression of profibrotic genes (*FN* and *α-SMA*) (Fig. 6c–f). Most importantly, Twist1 was high expression in patients with cirrhosis which was positively correlated with miR-214 level (Fig. 6g). In addition, luciferase reporter assay results confirmed that Twist1 regulate miR-214 expression through the E-box element within the promoter (Fig. 6h, i). All of these data suggested that Twist1 can indirectly affect fibrogenesis by upregulating miR-214.

Several reports showed that miR-214 participated in fibrosis. For example, miR-214 expression is upregulated in the renal fibrosis, and genetic deletion of miR-214 significantly attenuated kidney interstitial fibrosis induced by unilateral ureteral obstruction^30^. In the cardiac tissues, miR-214 is a sensitive marker of cardiac stress, and miR-214-deletion leads to increased fibrosis after ischemic injury^31^. However, the role of miR-214 in liver fibrosis is controversial according to the literatures. In this study, we took advantage of rat primary HSCs and human LX2 cells for in vitro studies, and found that the knockdown of miR-214 expression in HSCs and LX2 cells using antagomiR-214 resulted in cell morphological changes, decreased expression of profibrotic genes, and inhibition of cell proliferation. On the contrary, miR-214 overexpression promoted cell proliferation and induced profibrotic gene expression (Fig. 3 and Supplementary Fig. 2), thereby confirming that miR-214 takes part in the hepatic fibrogenesis.

The activation of HSCs and the development of hepatic fibrosis is regulated by many signaling pathways. Among these pathways, Hedgehog signaling is probably the most prominent direct autocrine and paracrine inducer of HSC activation^7–9^ and it is involved in fibrogenesis in many tissues. For example, the Sonic Hedgehog pathway has been shown to be activated in lung tissues affected by idiopathic pulmonary fibrosis^32^. Moreover, miR-378a-3p has been shown to suppress the activation of HSCs by targeting Gli3, and injection of miR-378a-3p mimics into mice was shown to ameliorate chronic liver injury induced by CCl_4_^18^. Using bioinformatics analysis, we identified Sufu, a well-known negative regulator of the Hedgehog pathway, as the potential target of miR-214. Sufu was reported to interact with Gli transcription factors to suppress their nuclear translocation and thereby inhibits Hedgehog signaling^10, 11^. In this study, we identified that miR-214 effectively repressed Sufu expression in HSCs and LX2 cells (Fig. 4a, b). Moreover, luciferase assay indicated that miR-214 inhibited Sufu expression by directly targeting the 3′-UTR of Sufu mRNA (Fig. 4e, f). Furthermore, we showed that knockdown of Sufu expression by siRNAs increased the expression of profibrotic genes, whereas Sufu overexpression had the opposite effects in both HSCs and LX2 cells. Most importantly, we observed reduced Sufu expression in clinical cirrhosis liver samples, which was negatively correlated with miR-214 expression (Fig. 5). Taken together, our data clearly demonstrate that miR-214 has an important role in HSC activation and hepatic fibrosis through affecting Sufu expression.

Manipulating the expression of dysregulated miRNAs could have anti-fibrotic effect in animal model. For example, Tu et al.^33^ reported that lentivirus-mediated ectopic expression of miR-101 in liver greatly reduced CCl_4_-induced liver fibrosis and Hyun et al.^18^ showed that systemic delivery of miR-378a-3p by nanoparticle technology significantly reduced hepatic damage. To evaluate the therapeutic potential of miR-214 repression for anti-fibrosis in vivo, chemically synthesized antagomiR-214 oligos were injected into CCl_4_-treated mice to downregulate miR-214 expression in the liver. Following treatment, Sufu expression was upregulated due to decreased miR-214 level in the CCl_4_/antagomiR-214 group when compared with that in the CCl_4_/NC-miR group. Masson’s trichrome and H&E staining clearly demonstrated that antagomiR-214 treatment effectively ameliorate liver fibrosis, implying that miR-214 is a therapeutic target for hepatic fibrosis treatment (Fig. 7).

**Fig. 8:**
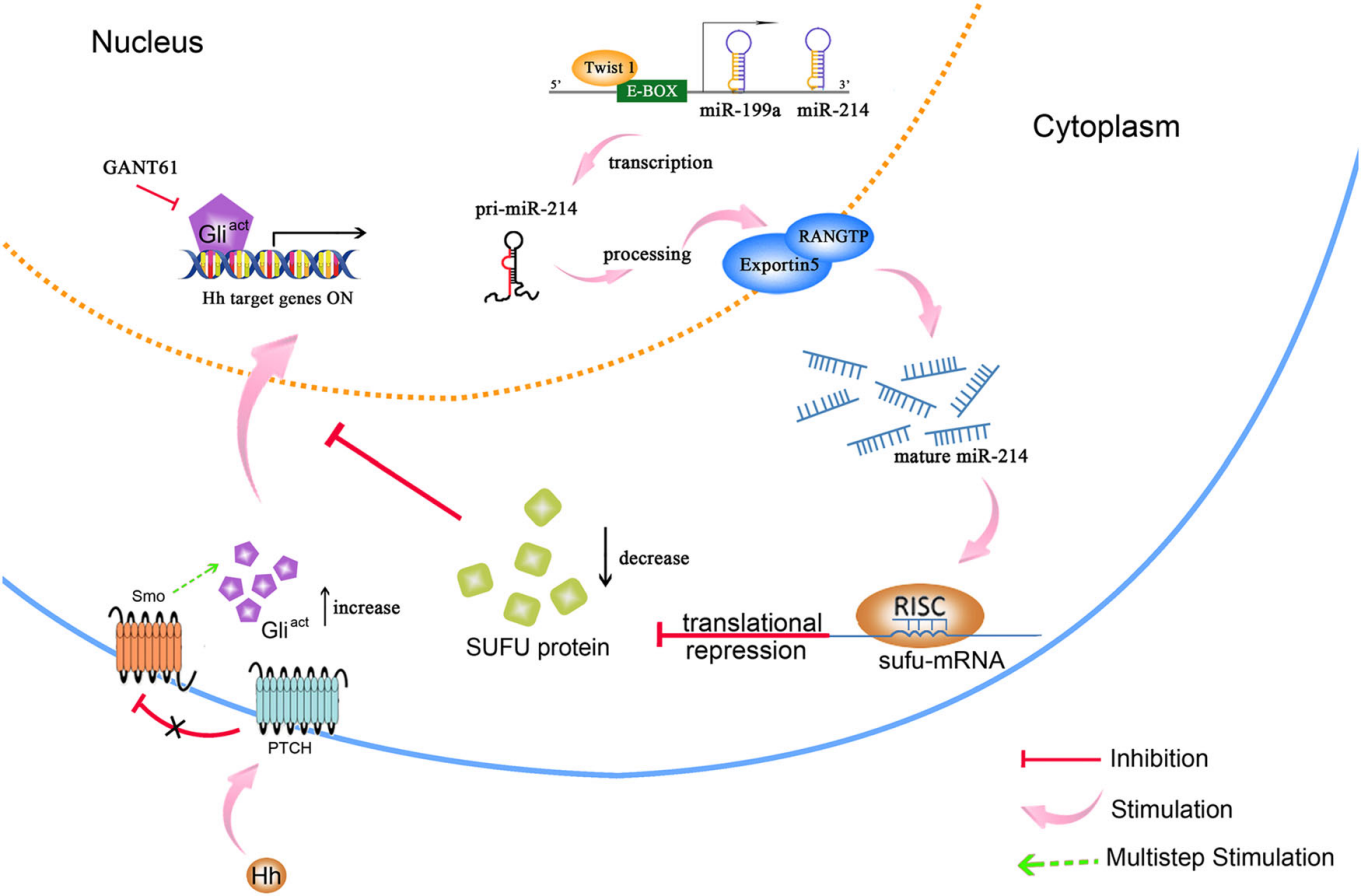
Model representing miR-214 regulates Sufu expression to participate in liver fibrogenesis. Transcription of *miR-214* gene is induced by the binding of Twist1 with the E-box element in the promoter region of the *miR-214* gene. Thus, increasing the expression of mature miR-214 which targets the 3′-UTR of Sufu to suppress its translation. Reduced expression of Sufu can no longer effectively inhibit Gli transcription factors, thereby accelerating the expression of Hedgehog signaling effectors and profibrotic genes in HSCs. GANT-61 is an inhibitor for Gli-induced transcript

In summary, we found that miR-214 expression is significantly upregulated during HSC activation and liver injury samples. Moreover, miR-214 expression is regulated by the transcriptional factor Twist1 through its binding to E-box element within the miR-214 promoter. Functional studies demonstrated that miR-214 plays a pivotal role in hepatic fibrosis by regulating Sufu expression, and knockdown of miR-214 expression by antagomiRs effectively alleviate liver fibrosis in CCl_4_-treated mice. These findings indicate that miR-214 has great potential to be used as a biomarker of hepatic fibrosis and a therapeutic target for treating liver diseases (Fig. 8).

## Materials and Methods

### Isolation, culture, and identification of HSCs

Primary HSCs were isolated from normal male Sprague–Dawley rats (Animal Center of Sichuan University, Chengdu, China) by in situ pronase/collagenase (Roche Life Science, Indianapolis, IN, USA) perfusion followed by subsequent density gradient centrifugation, as described previously^34^. The obtained cell purity was more than 95%. The isolated HSCs were then cultured in low glucose Dulbecco’s modified Eagle's medium (DMEM) (Gibco, USA), supplemented with 20% fetal bovine serum (FBS) (Biological Industries, Israel), and maintained in 37 °C incubator with 5% CO_2_. Culture medium was replaced every 48 h.

Immortalized human HSC cell line LX2 (Procell, Wuhan, China) was cultured in high glucose DMEM (Gibco, USA), supplemented with 10% FBS and maintained in 37 °C incubator with 5% CO_2_.

### Microarray analysis

Total RNAs were isolated from quiescent (cultured for 1 day) and activated (cultured for 12 days) primary HSCs, respectively, using TRIzol reagent (Life Technologies, Grand Island, NY, USA) and purified using mirVana™ miRNA Isolation Kit (Life Technologies, Grand Island, NY, USA). The purified total RNAs were labeled and hybridized with microchip using miRNA Complete Labeling and Hyb Kit (Agilent Technologies, Santa Clara, CA, USA). Hybridization signals were detected by DNA microarray scanner G2565CA (Agilent Technologies, Santa Clara, CA, USA), and the scanned images were analyzed using Agilent Feature Extraction software (version 10.7).

### Liver histopathology and fibrosis measurement

Excised rat or mouse liver tissues were fixed in 4% paraformaldehyde, embedded in paraffin, and sectioned, followed by staining with H&E (Beyotime, Shanghai, China), trichrome (Masson) stain kit (Baso Diagnostics Inc., Zhuhai, China), or ORO staining (Beyotime, Shanghai, China). For semi-quantitative analysis of severity of liver fibrosis, five randomly selected fields per liver section from each animal were recorded, and Masson-stained fibrosis areas were quantified using the ImagePro Plus 6.0 software (Media Cybernetics, Bethesda, MD, USA).

Immunohistochemical staining was performed on the sections from the paraffin-embedded mouse liver tissues. Briefly, after deparaffinization, hydration, and antigen retrieval, samples were incubated overnight at 4 °C with a primary antibody against α-SMA (ab7817; Abcam) (1:100) and then with a biotinylated secondary antibody. The expression of α-SMA was visualized by 3,3′-diaminobenzidine tetrahydrochloride staining.

### Clinical liver samples collection

Liver tissues were obtained from patients with cirrhosis and healthy subjects from West China Hospital of Sichuan University. The tissues were rapidly frozen in liquid nitrogen following surgical resection. All specimens were collected upon obtaining informed consent from all patients, and the study was approved by the Human Ethics Committee of Sichuan University.

### Cell transfection and treatment

Rat HSCs or human LX2 cells were transfected with miRNA mimics, antagomiR, or siRNA (GenePharma, Shanghai, China) using Lipofectamine 2000 (Invitrogen, Carlsbad, CA, USA) at a final concentration of 100 nM for 48 h. Total RNAs and proteins were collected for RT-qPCR and Western blotting, respectively. To study the effect of Gli on fibrogenesis, HSCs and LX2 were treated with 5 μM of Gli inhibitor GANT-61 (Selleck, Boston, MA, USA) for 24 h.

### RT-qPCR

Total RNAs were isolated using TRIzol reagent. For quantification of miRNA expression, cDNA was synthesized using Reverse Transcription Kit (GeneCopoeia, Rockville, MD, USA), and quantified using Hairpin-it^TM^ miRNA RT-PCR Quantitation Kit (GenePharma, Shanghai, China). For mRNA quantification, reverse transcription was performed using High Capacity cDNA Reverse Transcription Kit (Applied Biosystems, Foster City, CA, USA), and mRNA levels were measured with SYBR Green Mix (Life Technologies, Grand Island, NY, USA) using the primers listed in Supplementary Table 1.

### Western blot analysis

Protein was extracted with RIPA lysis buffer (50 mM Tris-HCl, pH 7.4, 100 mM 2-mercaptoethanol, 2% sodium dodecyl sulfate (SDS), 10% glycerol) containing protease inhibitors and phosphatase inhibitors, and protein concentration was measured by Pierce BCA Protein Assay Kit (Thermo Scientific, USA). Following separation by SDS-polyacrylamide gel electrophoresis, proteins were transferred onto PVDF membrane (Millipore, Billerica, MA, USA). Primary antibodies used in this study were as follows: rabbit anti-Sufu (C54G2; Cell Signaling Technologies, Danvers, MA, USA), mouse anti-α-SMA (ab7817; Abcam, Cambridge, UK), rabbit anti-FN (ab2413; Abcam, Cambridge, UK), mouse anti-myc tag (Santa Cruz Biotechnology, CA, USA), and rabbit anti-β-actin (13E5; Cell Signaling Technologies, Danvers, MA, USA). Horseradish peroxidase-conjugated anti-rabbit (Sigma, USA) or anti-mouse IgG (GE, USA) was used as secondary antibody. Protein bands were detected using SuperSignal West Dura Extended Duration Substrate (Thermo Scientific, USA), and the densities of protein bands were measured using the Quantity One software.

### Plasmid construction

The primers for plasmid construction are listed in Supplementary Table 2. For overexpression study, the sequence encoding full-length Sufu and Twist1 was amplified and cloned into the lentiviral vector pCDH-CMV-MCS-EF1-copGFP.

The 3′-UTR region of rat and mouse Sufu mRNA containing the potential miR-214 binding site was cloned into pmirGLO Dual-Luciferase miRNA Target Expression Vector (Promega, Madison, WI, USA). Mutations within potential miR-214 binding sites were introduced by QuikChange Site-Directed Mutagenesis Kit (Life Technologies, Grand Island, NY, USA).

The miR-214 promoter region was amplified by PCR from the genomic DNA of primary rat HSCs, and the resultant fragments were cloned into pGL4.27 vector (Promega, Madison, WI, USA) at *Xho*I and *Hind*III (New England Biolabs, Ipswich, MA, USA) sites. The mutant miR-214 promoter containing 2-base point mutation (CATCTG → CACGTG) in the E-box site was generated and verified by DNA sequencing.

### Cell cycle assay

Cell Cycle Analysis Kit (Beyotime, Shanghai, China) was utilized to analyze the cell cycle. Cells were fixed in 70% ethanol in PBS at −20 °C for 24 h and then treated with 50 μg/ml RNase A at 37 °C for 30 min. Then, the cells were labeled with 50 μg/ml propidium iodide staining buffer in dark at 4 °C for 30 min. The analyses were performed on BD LSR flow cytometer (BD Biosciences, San Jose, CA, USA)

### Luciferase reporter assay

For examining whether miR-214 directly target Sufu mRNA, pMir-Report-sufu reporter plasmids were co-transfected with miR-214 mimics or scramble into HEK 293T cells using Lipofectamine 2000 for 24 h. After transfection, both firefly and Renilla luciferase activities were measured by Dual-Luciferase Assays Kit (Promega, Madison, WI, USA). The firefly luciferase activity was normalized to Renilla luciferase activity.

For investigating whether Twist1 binds to the promoter of miR-214 gene, the recombinant pGL4.27 plasmid containing wild-type or mutant miR-214 gene promoter was co-transfected into HEK 293T cells with Twist1 expression plasmid and pRL-TK containing Renilla luciferase reporter gene. Then, the firefly and Renilla luciferase activities were measured at 24 h after transfection.

### Animal experiments

Adult male Sprague–Dawley rats (6–8 weeks, 200–300 g) and male C57BL/6 mice (6–8 weeks, 20–22 g) were provided by the Experimental Animal Center of Sichuan University. All animal studies were approved by the Medical Ethics Committee of the West China Hospital of Sichuan University.

For CCl_4_-induced liver fibrosis model, male rats were injected subcutaneouly with 0.3 ml of olive oil or CCl_4_/olive oil (3:2, v/v)/100 g body weight twice a week. At different times, rats were sacrificed to assess the success of the model building.

To investigate the effect of miR-214 antagomiR on hepatic fibrosis in vivo, 15 male C57BL/6J mice were randomized into three groups: Olive oil group, CCl_4_/NC group, and CCl_4_/antagomiR-214 group. Mice were injected intraperitoneally with 0.1 ml of olive oil or CCl_4_/olive oil (1:3, v/v)/100 g twice a week for 5 weeks to obtain the liver fibrosis mice or control mice. Treatments started at the 10th day after CCl_4_ injections, and animals were received 0.2 ml vehicle or antagomiR-214 twice a week at 62.5 nmol/body dose level via tail vein injection. Mice were sacrificed at the end of the 5-week treatment, and the livers were collected for further analysis.

For NASH model, healthy male C57BL/6J mice were fed with high-fat diet (Research Diet, New Brunswick, NJ, USA) and control group mice were fed with normal diet. These mice were sacrificed after 20 weeks modeling, and the livers were collected for further analysis.

### Statistical analysis

Data are shown as the mean ± standard error of the mean (SEM) and are representative of at least three independent experiments. Statistical analysis among groups using the two-tailed Student's *t* test, *P* *<* 0.05 was considered statistically significant.

## Electronic supplementary material

Supplementary Table

Supplementary figure 1

Supplementary figure 2

Supplementary figure 3

Supplementary figure legends

## References

[1] Friedman SL (2010). Evolving challenges in hepatic fibrosis. Nat. Rev. Gastroenterol. Hepatol..

[2] Aleffi S (2005). Upregulation of proinflammatory and proangiogenic cytokines by leptin in human hepatic stellate cells. Hepatology.

[3] Tsuchida T, Friedman SL (2017). Mechanisms of hepatic stellate cell activation. Nat. Rev. Gastroenterol. Hepatol..

[4] Ding N (2013). A vitamin D receptor/SMAD genomic circuit gates hepatic fibrotic response. Cell.

[5] Cho IJ (2010). E-cadherin antagonizes transforming growth factor β1 gene induction in hepatic stellate cells by inhibiting RhoA-dependent Smad3 phosphorylation. Hepatology.

[6] Son G, Hines IN, Lindquist J, Schrum LW, Rippe RA (2009). Inhibition of phosphatidylinositol 3-kinase signaling in hepatic stellate cells blocks the progression of hepatic fibrosis. Hepatology.

[7] Yang L (2008). Sonic hedgehog is an autocrine viability factor for myofibroblastic hepatic stellate cells. J. Hepatol..

[8] Chen Y (2012). Hedgehog controls hepatic stellate cell fate by regulating metabolism. Gastroenterology.

[9] Scales SJ, de Sauvage FJ (2009). Mechanisms of Hedgehog pathway activation in cancer and implications for therapy. Trends Pharmacol. Sci..

[10] Hui CC, Angers S (2011). Gli proteins in development and disease. Annu. Rev. Cell Dev. Biol..

[11] Kogerman P (1999). Mammalian suppressor-of-fused modulates nuclear cytoplasmic shuttling of GLI-1. Nat. Cell Biol..

[12] Peng Y, Croce CM (2016). The role of microRNAs in human cancer. Signal Transduct. Target Ther..

[13] Drusco A, Croce CM (2017). MicroRNAs and cancer: a long story for short RNAs. Adv. Cancer Res..

[14] Chen CZ, Li L, Lodish HF, Bartel DP (2004). MicroRNAs modulate hematopoietic lineage differentiation. Science.

[15] Rottiers V, Näär AM (2012). MicroRNAs in metabolism and metabolic disorders. Nat. Rev. Mol. Cell. Biol..

[16] Roderburg C (2011). MicroRNA profiling reveals a role for miR-29 in human and murine liver fibrosis. Hepatology.

[17] Lakner AM (2012). Inhibitory effects of microRNA-19b in hepatic stellate cell-mediated fibrogenesis. Hepatology.

[18] Hyun J (2016). MicroRNA-378 limits activation of hepatic stellate cells and liver fibrosis by suppressing Gli3 expression. Nat. Commun..

[19] Ogawa T (2012). MicroRNA-221/222 upregulation indicates the activation of liver fibrosis. Gut.

[20] Shi J, Aisaki K, Ikawa Y, Wake K (1998). Evidence of hepatocyte apoptosis in rat liver after the administration of carbon tetrachloride. Am. J. Pathol..

[21] Musso G, Cassader M, Gambino R (2016). Non-alcoholic steatohepatitis: emerging molecular targets and therapeutic strategies. Nat. Rev. Drug Discov..

[22] Bataller R, Brenner DA (2015). Liver fibrosis. J. Clin. Invest..

[23] Long H (2015). microRNA-214 promotes epithelial-mesenchymal transition and metastasis in lung adenocarcinoma by targeting the suppressor-of-fused protein (Sufu). Oncotarget.

[24] Lee YB (2009). Twist-1 regulates the miR-199a/214 cluster during development. Nucleic Acids Res..

[25] Noetel A, Kwiecinski M, Elfimova N, Huang J, Odenthal M (2012). microRNA are central players in anti- and profibrotic gene regulation during liver fibrosis. Front. Physiol..

[26] Maubach G, Lim MC, Chen J, Yang H, Zhuo L (2011). miRNA studies in vitro and in vivo activated hepatic stellate cells. World J. Gastroenterol..

[27] Chen L (2014). Epigenetic regulation of connective tissue growth factor by microRNA-214 delivery in exosomes from mouse or human hepatic stellate cells. Hepatology.

[28] Bridges RS (2009). Gene expression profiling of pulmonary fibrosis identifies Twist1 as an antiapoptotic molecular “rectifier” of growth factor signaling. Am. J. Pathol..

[29] Lee YH (2016). Elevation of Twist expression by arecoline contributes to the pathogenesis of oral submucous fibrosis. J. Formos. Med. Assoc..

[30] Denby L (2014). MicroRNA-214 antagonism protects against renal fibrosis. J. Am. Soc. Nephrol..

[31] Aurora AB (2012). MicroRNA-214 protects the mouse heart from ischemic injury by controlling Ca^2+^ overload and cell death. J. Clin. Invest..

[32] Bolanos AL (2012). Role of sonic Hedgehog in idiopathic pulmonary fibrosis. Am. J. Physiol. Lung Cell. Mol. Physiol..

[33] Tu X (2014). MicroRNA-101 suppresses liver fibrosis by targeting the TGFβ signaling pathway. J. Pathol..

[34] Weiskirchen R, Gressner AM (2005). Isolation and culture of hepatic stellate cells. Methods Mol. Med..

