# Supplementary material for: MicroRNA-214 promotes hepatic stellate cell activation and liver fibrosis by suppressing Sufu expression
Source: Cell Death Dis. 2018 Jun 18;9(7):718. doi: 10.1038/s41419-018-0752-1 (PMC6006298; doi:10.1038/s41419-018-0752-1)
Supplement: Supplementary file 1 — Supplementary Table [file 41419_2018_752_MOESM1_ESM.docx]

**Supporting Table 1. Primers information for RT-qPCR analysis**

| Primer name | Forward Primer (5'-3') | Reverse Primer (5'-3') |
| --- | --- | --- |
| GAPDH(Rat) | cccatcaccatcttccaggag | gttgtcatggatgaccttggc |
| β-ACTIN(Rat) | actatcggcaatgagcggttc | atgccacaggattccataccc |
| α-SMA(Rat) | ccgagatctcaccgactacc | tccagagcgacatagcacag |
| FN(Rat)  SUFU(Rat/Mouse/Human) | gagagatctggaggtcat  gaccctggttacaaattctgttg | gggtgacacctgagttgaa  cagctgtactctttgggaagt |
| TWIST1(Rat/Mouse/Human) | actccaagatggcaagctg | tagtgggacgcggacat |
| COL1α1(Rat) | acgtcctggtgaagttggtc | tccagcaataccctgaggtc |
| COL1α1(Mouse) | acgtcctggtgaagttggtc | ccacgtctcaccattgggg |
| GAPDH(Mouse) | cgacttcaacagcaactcccactcttcc | tgggtggtccagggtttcttactcctt |

**Supporting Table 2. Primers information for plasmid construction**

| Primer name | Primer (5'-3') |
| --- | --- |
| Rat sufu wt 3’UTR F | tccgctcgagggtggctccatacttggg |
| Rat sufu wt 3’UTR R | tactgtcgacctctggaagaaaccagagc |
| Rat Sufu mut 3’UTR F | ggaatgtctaaaatcctattgggtccagacccccatcac |
| Rat Sufu mut 3’UTR R  Mouse sufu wt 3’UTR F  Mouse sufu wt 3’UTR R  Mouse sufu mut 3’UTR F  Mouse sufu mut 3’UTR R  E-box wt promoter F  E-box wt promoter R  E-box mut promoter F  E-box mut promoter R  Rat Sufu F  Rat Sufu R  Human sufu F Human sufu R  myc Twist1 F  myc Twist1 R | Gtgatgggggtctggacccaataggattttagacattcc tccgctcgagtctttgcagactggtggttac  tactgtcgac tgagtttagccactctcctaga  caggaatgtctaaactcctattgggaccagaccctgtc  gacagggtctggtcccaataggagtttagacattcctg  tccgctcgaggaagtttgcaggcttgaca  tactaagcttgggctgtgatttccagtctt  gcttaacattacacgaaaacacgtggtaccattttatgcacag  ctgtgcataaaatggtaccacgtgttttcgtgtaatgttaagc  gtcatctagagccaccatggcggagctgaggcct  tactgaattcctagtgcagcggactgtcga  gtcatctagagccaccatggcggagctgcggcct  tactggatccctagtgtagcggactgtcga  gtcatctagagccaccatggaacaaaaactcatctcagaagaggatctgatgatgcaggacgtgtccatactgaattcctagtgggacgcggacat |

F: forward primer; R: reverse primer; wt: wild type; mut: mutant
