# Supplementary figures and images for: MicroRNA-214 promotes hepatic stellate cell activation and liver fibrosis by suppressing Sufu expression
Source: Cell Death Dis. 2018 Jun 18;9(7):718. doi: 10.1038/s41419-018-0752-1 (PMC6006298; doi:10.1038/s41419-018-0752-1)

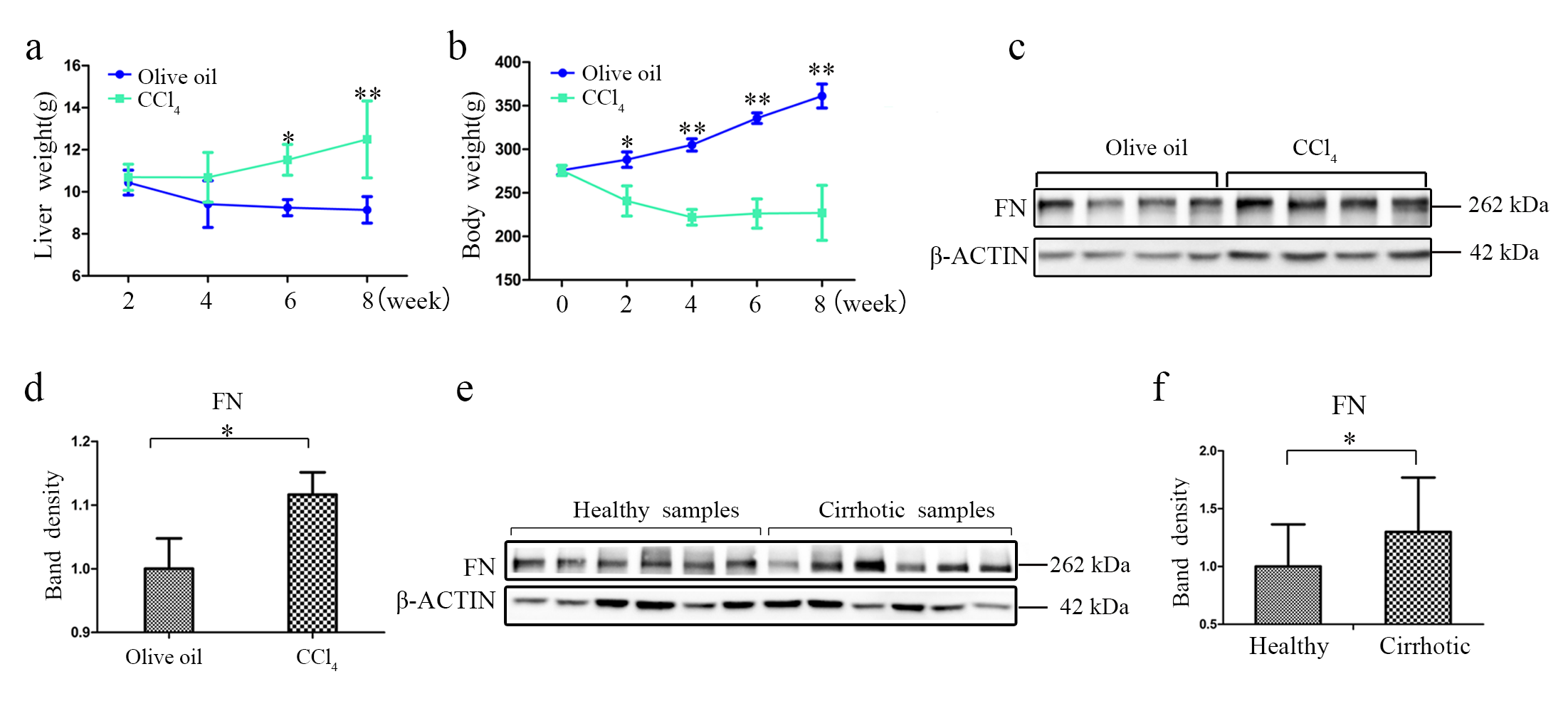

Supplement: Supplementary file 2 — Supplementary figure 1 [file 41419_2018_752_MOESM2_ESM.tif]

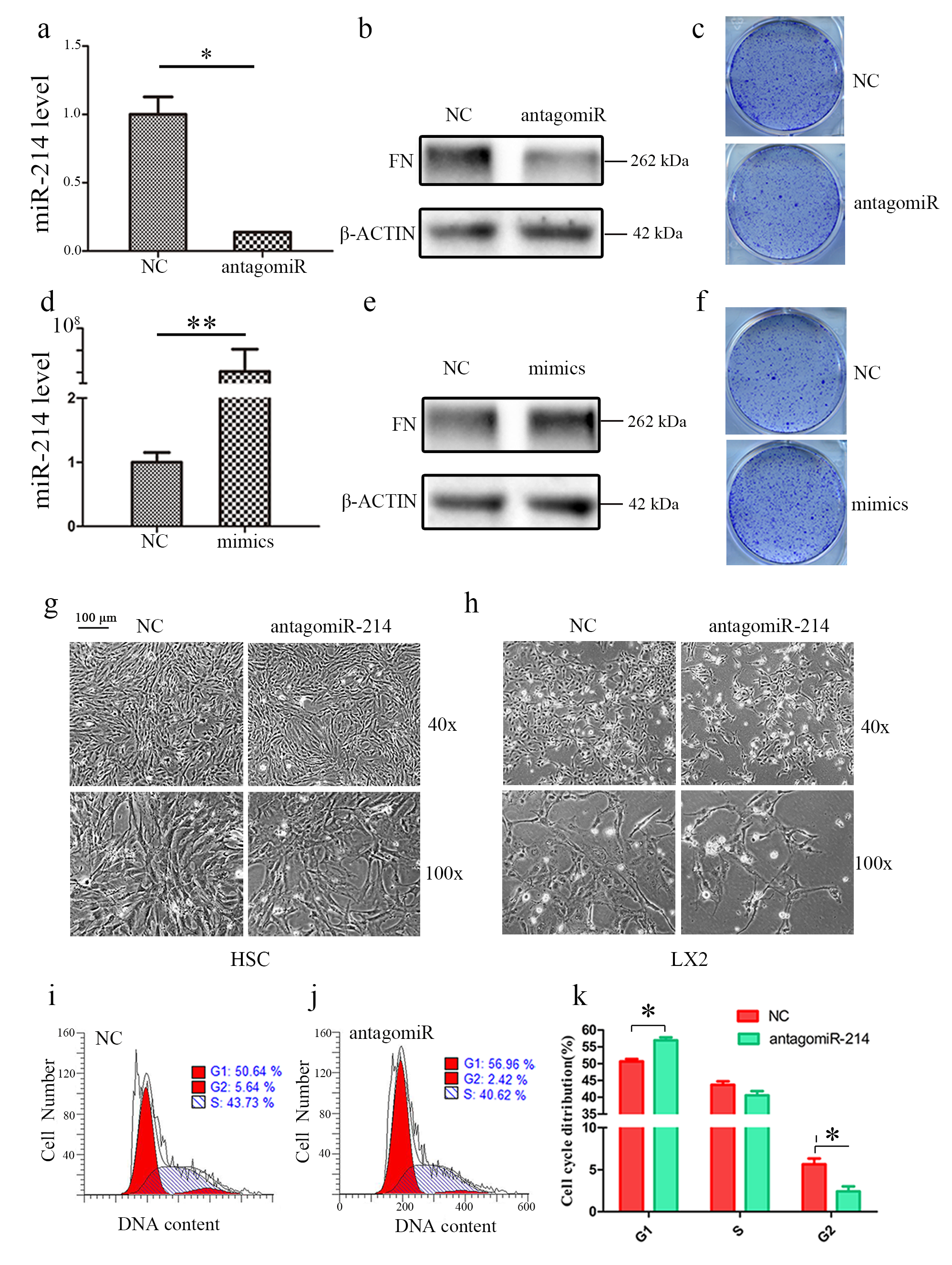

Supplement: Supplementary file 3 — Supplementary figure 2 [file 41419_2018_752_MOESM3_ESM.tif]

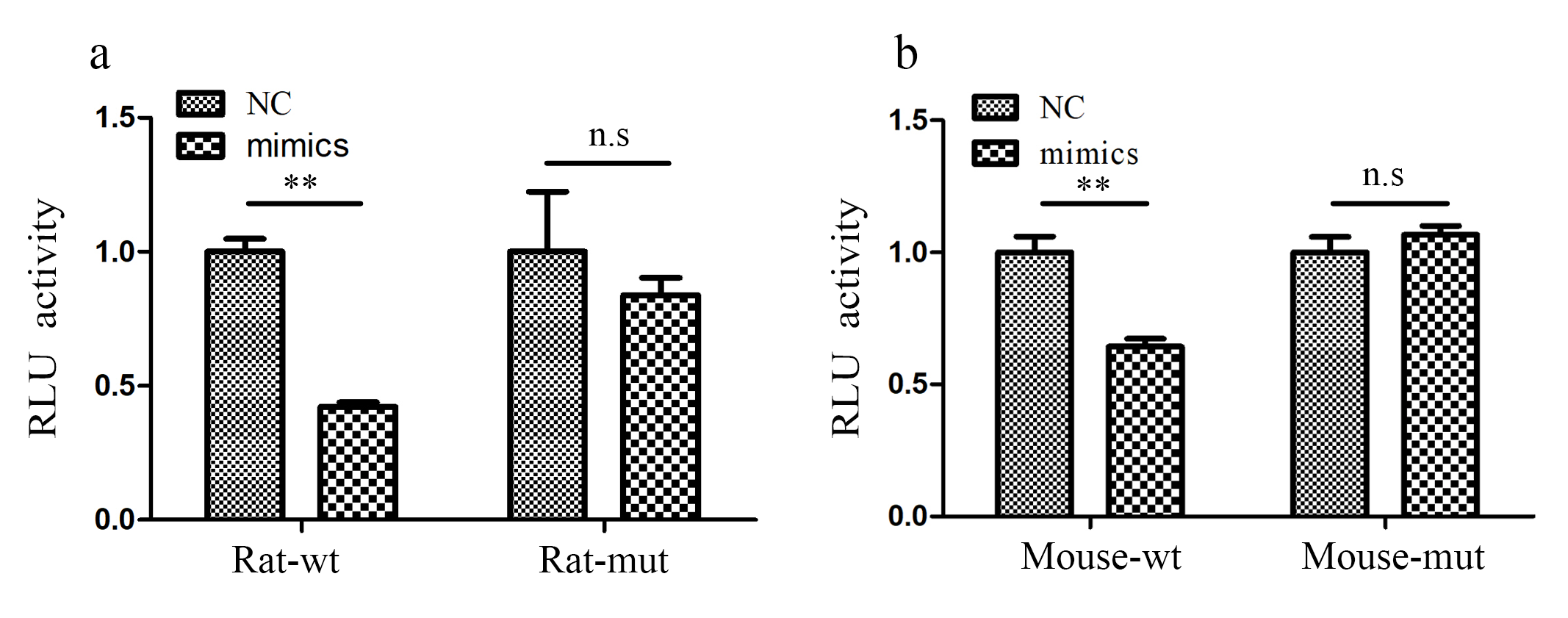

Supplement: Supplementary file 4 — Supplementary figure 3 [file 41419_2018_752_MOESM4_ESM.tif]
