# Supplementary material for: MicroRNA-214 promotes hepatic stellate cell activation and liver fibrosis by suppressing Sufu expression
Source: Cell Death Dis. 2018 Jun 18;9(7):718. doi: 10.1038/s41419-018-0752-1 (PMC6006298; doi:10.1038/s41419-018-0752-1)
Supplement: Supplementary file 5 — Supplementary figure legends [file 41419_2018_752_MOESM5_ESM.docx]

**Supplementary Fig. 1. Liver injuries in CCl4-treated rats and human cirrhosis samples**(a) Body weight and (b) liver weight of rats treated with CCl4 or olive oil for 2, 4, 6, and 8 weeks. (c) FN expression in rats treated with CCl4 or olive oil for 8 weeks and (d) Cumulative densitometric analyses of FN expression using Western blotting in rat liver samples. (e) Expression of FN in livers of cirrhosis patients or healthy controls and (f) Cumulative densitometric analyses of FN expression using western blotting in human liver samples. Relative expression levels are shown as the means ± s.e.m. (unpaired two-sample Student’s t-test, * P < 0.05 and ** P < 0.01).

**Supplementary Fig. 2. miR-214 regulates fibrotic gene expression and promotes LX2 cell proliferation**

(a) Knockdown of miR-214 expression by transfection with antagomiR-214 or NC-miR in LX2 cells and (b) the protein level of FN was detected by Western blotting. (c) Proliferation of LX2 cells was measured using colony formation assay after transfection with antagomiR-214. (d) miR-214 overexpression in LX2 cells by mimics or NC-miR and (e) the protein expression of FN was detected by Western blotting. (f) Proliferation of LX2 cells was measured using colony formation assay after transfection with miR-214 mimics. (g) Morphology of rat HSCs and (h) LX2 cells after transfection with antagomiR-214 or NC-miR at 40x or 100x magnification. (i-k) Knockdown of miR-214 in rat HSCs increased the proportion of cells in G1 phase but decreased the proportion of cells in G2 phase as examined using flow cytometry. Relative expression levels are shown as the means ± s.e.m. obtained from triplicate experiments (unpaired two-sample Student’s t-test, * *P* < 0.05 and ** *P* < 0.01).

**Supplementary Fig. 3. miR-214 directly targets 3’-UTR of Sufu mRNA in HSCs**

Dual-luciferase reporter assays were performed to verify the binding between miR-214 and rat sufu mRNA (a) or between miR-214 and mouse sufu mRNA (b). HSCs were co-transfected with luciferase reporter with either wild-type (wt) or mutant (mut) 3’-UTR of Sufu mRNA and either miR-214 mimics or negative control (NC). Relative luciferase activity (RLU) was shown as the means ± S.E.M. obtained from triplicate experiments (two-sample Student’s t-test, ** *P* < 0.01)
